# Potential Nutraceutical Benefits of In Vivo Grown Saffron (*Crocus sativus* L.) As Analgesic, Anti-inflammatory, Anticoagulant, and Antidepressant in Mice

**DOI:** 10.3390/plants9111414

**Published:** 2020-10-22

**Authors:** Asif Khan, Nur Airina Muhamad, Hammad Ismail, Abdul Nasir, Atif Ali Khan Khalil, Yasir Anwar, Zahid Khan, Amjad Ali, Rosna Mat Taha, Baker Al-Shara, Sara Latif, Bushra Mirza, Yousef Abdal Jalil Fadladdin, Isam Mohamed Abu Zeid, Saed Ayidh Al-Thobaiti

**Affiliations:** 1 Institute of Biological Sciences, Faculty of Science, University of Malaya, 50603 Kuala Lumpur, Malaysia; asif.khan.qau@gmail.com (A.K.); rosnataha2020@gmail.com (R.M.T.); Bakearbio@yahoo.com (B.A.-S.); 2 Department of Biochemistry and Biotechnology, University of Gujrat, Gujrat 50700, Pakistan; hammad.ismail@uog.edu.pk; 3 Department of Molecular Science and Technology, Ajou University, Suwan 16499, Korea; anasirqau@gmail.com; 4 Department of Biological Sciences, National University of Medical Sciences, Rawalpindi 46000, Pakistan; Atif.ali@numspak.edu.pk; 5 Department of Biological Sciences, Faculty of Science, King Abdulaziz University, P.O. Box 54229, Jeddah, Saudi Arabia; yanwarulhaq@kau.edu.sa (Y.A.); yfadladdin@kau.edu.sa (Y.A.J.F.); abuzeidmm@yahoo.com (I.M.A.Z.); 6 Department of Pharmacognosy, Faculty of Pharmacy, Federal Urdu University of Arts Science and Technology, Karachi 75300, Pakistan; zahidkhan@fuuast.edu.pk; 7 Department of Botany, University of Malakand, Khyber Pakhtunkhwa 18800, Pakistan; amjad1990.aa48@gmail.com; 8 Department of Biochemistry, Quaid-i-Azam University, Islamabad 45320, Pakistan; saralatifawan@gmail.com (S.L.); bushramirza@qau.edu.pk (B.M.); 9 Lahore College for Women University, Lahore 54000, Pakistan; 10 Department of Biology, University College Turabah, Taif University, Taif 21995, Saudi Arabia; saed@tu.edu.sa

**Keywords:** *Crocus sativus*, in vivo, carrageenan, analgesic, antidepressant, anticoagulant

## Abstract

*Crocus sativus*, a medicinally important herbaceous plant, has been traditionally used to cure coughs, colds, insomnia, cramps, asthma, and pain. Moreover, the therapeutic applications of saffron include its immunomodulatory and anticancer properties. The current experimental analysis was performed to explore the potential nutraceutical efficacy of corm, leaf, petal, and stigma of saffron ethanolic extracts as analgesic, anti-inflammatory, anticoagulant, and antidepressant using hot plate, carrageenan-induced paw edema, capillary tube and forced swim test, respectively in mice. The results indicated that among all the extracts, stigma ethanolic extract (SEE) represented maximum latency activity (72.85%) and edema inhibition (77.33%) followed by petal ethanolic extract (PEE) with latency activity and edema inhibition of 64.06 and 70.50%, respectively. Corm ethanolic extract (CEE) and leaf ethanolic extract (LEE) displayed mild analgesic activity of 22.40% and 29.07%, respectively. Additionally, LEE (53.29%) and CEE (47.47%) exhibited mild to moderate response against inflammation. The coagulation time of SEE (101.66 s) was almost equivalent to the standard drug, aspirin (101.66 s), suggesting a strong anticoagulant effect followed by PEE (86.5 s). LEE (66.83 s) represented moderate inhibitory effect on coagulation activity while CEE (42.83 s) showed neutral effect. Additionally, PEE and SEE also expressed itself as potential antidepressants with immobility time ≤76.66 s, while CEE (96.50 s) and LEE (106.83 s) indicated moderate to mild antidepressant efficacy. Based on the in vivo activities, saffron extract, particularly SEE and PEE, can be used as a potential nutraceutical and therapeutic agent due to its significant pharmacological activities.

## 1. Introduction

In recent times, traditional medicinal plants are gaining much attention in pharmaceutical industries and served as a part of the primary medical emergencies in the treatment of numerous diseases. *Crocus sativus*, or saffron, is one such stemless herbaceous plant grown as a source of its spice for nearly 3600 years and belongs to the family Iridaceae. Saffron is geographically distributed in Irano-Touranian regions, East Asia, and Mediterranean climates and largely cultivated in countries like Iran, Afghanistan, India, France, Turkey, Italy, Spain, and Morocco [1]. Current estimates for total world annual production of saffron are 418 t per year [2]. Iran, the world’s largest producer of saffron with around 90,000 ha harvest area and 336 t annual yield, is said to produce more than 90% of the total saffron produced worldwide [3].

**Figure 1:**
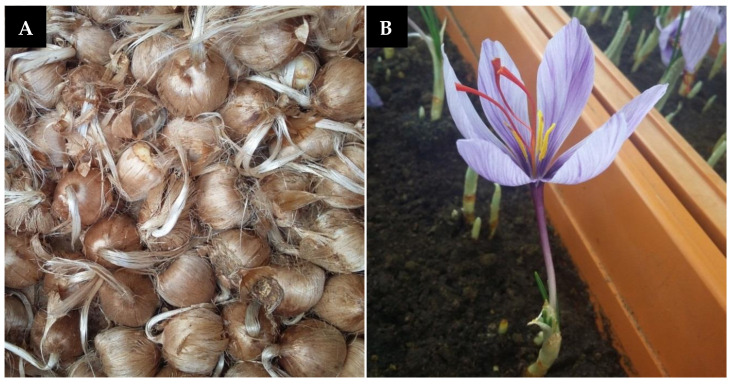
Main components of saffron (*Crocus sativus*), (**A**) corms (**B**) flower.

Saffron plant comprises of the corm, foliar structure, and floral organs as the main parts (Figure 1). Commercially, saffron is a dietary spice obtained from three red pungent stigmatic lobes of the *Crocus sativus* flower. According to an estimate, it takes around 60,000 Crocus flowers to produce just 1 kg of this unique spice, which equates to 370 to 470 h of work [4] and needs a cultivation area of approximately 2000 m^2^ [5]. At a retail price of almost US$1000–10,000 per kg, saffron is considered the costliest spice of the world [6]. Saffron stigma contains more than 150 phytochemical ingredients, belonging to various divisions of secondary metabolites such as carotenoids, flavonoids, terpenoids, and anthocyanin. Amongst the 50 constituents identified so far, apocarotenoids, including crocin, crocetin, picrocrocin, and safranal, emerge as the major constituents of saffron stigma, primarily responsible for imparting a distinct colorant, flavor, and aroma to the spice [7]. These compounds are derived from lipophilic carotenoid, zeaxanthin by oxidative cleavage. Moreover, a series of flavonoids (all glycosidic derivatives of kaempferol) have also been characterized in the stigma and petals of saffron, which, together with picrocrocin, give the characteristic bitter feature to this spice [8]. Furthermore, some anthraquinones and anthocyanin secondary components are also reported to be extracted from corms and petals of saffron [9].

Conventionally, saffron has a long history of being used as a spice and food additive [10]. Besides its use in culinary [11] and cosmetic preparation [12], saffron has been used as a folk remedy for coughs, colds, insomnia, cramps, asthma and bronchospasms, liver disease, pain, and epilepsy [2]. Saffron tea has been reputed to be a potential complementary treatment for psoriasis in medical nutrition therapy [13]. Many pharmacological studies have shown that this plant and its phytochemicals have emerged as nutraceutical elements, endowed with beneficial effects on health showing antibacterial [14], antioxidant, cytotoxic [15,16], antifungal [17], immunomodulatory [18,19], anti-mutagenic [20], aphrodisiac [21], antitussives [22,23], and antiplatelet effects [24]. Traditional and modern biomedical reports have also proved saffron to cure coronary artery diseases [25], respiratory diseases [26], menstrual disorders [27,28], and neurodegenerative disorders [29]. All these desirable properties of saffron have been attributed to the stigmas, whereas other parts of the plant have been much less studied. Nevertheless, a large quantity of saffron by-products is produced during the stigmatization process with little commercial value but thrown away after harvesting. According to an estimate, approximately 1500 kg of leaves, 350 kg of petals, and several hundred cormlets too small for cultivation or biologically and physically damaged to be regrown are rejected to get spice of only 1 kg [30]. However, this biomass contains a multitude of phytochemical ingredients whose exploitation would significantly enhance the sustainability and profitability of saffron yield. These saffron based by-products need to be tested to assess its pharmacological applications.

Bioactive compounds from petals have shown antinociceptive, anti-inflammatory [31], cytotoxic, antimicrobial, antioxidant, antidiabetic [32], antiobesity [33], antidepressant [34], anticancer, antityrosinase properties, as well as reduce blood pressure and contractility [35]. Several phenolic compounds from saffron leaves have been identified, showing antibacterial [36,37], anticancer [30], antioxidant, and metal ion chelating activities [37,38]. Likewise, bioactive constituents in saffron corm, such as proteoglycans, revealed to have a cytolytic effect on human tumor and plant cells and triterpenoid saponins on fungicidal and anticancer activities [30]. Polyphenols showing antibacterial, radical scavenging activity [39,40], and a mannan-binding lectin [41] have also been investigated in saffron corms. The existing experimental analysis aimed to explore the anti-inflammatory, analgesic, anticoagulant, and antidepressant potentials of various extracts of *Crocus sativus* L. in mice by carrageenan-induced paw edema test, hot plate assay, capillary method, and forced swim test, respectively. Up to now, ethanolic extracts of stigma, petals, corms, and leaves of in vivo cultivated saffron have not been previously studied for their nutraceutical properties against analgesia, inflammation, coagulation, and depression.

## 2. Results

### 2.1. Acute Toxicity Study

During the 7-days study, none of the orally administered saffron ethanolic extracts showed any mortality in the animals treated with 800 mg/kg. Furthermore, tested samples did not produce any notable behavioral changes in mice during the observation period.

### 2.2. Hot Plate Analgesic Test

**Figure 2:**
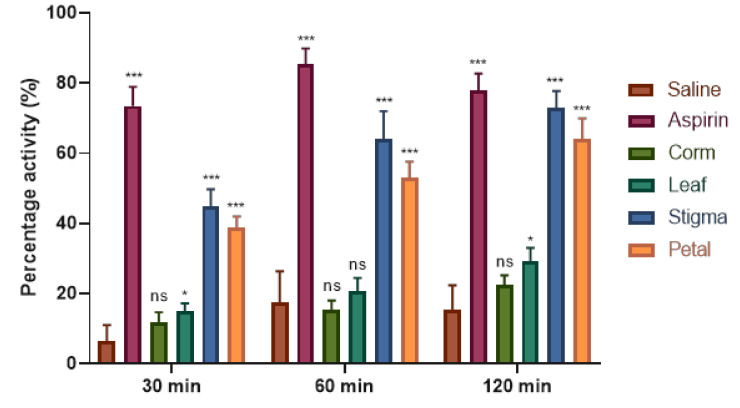
Percentage analgesia of C*. sativus* ethanolic extracts at selected time period in mice. Each value is represented as mean ± S.D. Where, * *p* < 0.05, ** *p* < 0.01, *** *p* < 0.001 statistically significant relative to control (saline).

The test is based on the use of thermal stimuli to determine the effect of analgesics. For this purpose, an easy and cost-effective method, the hot plate analgesic test, was performed. Results of saffron ethanolic extracts presented in the form of latency time indicated that all the extracts showed significant (*p* < 0.05) analgesic effect in a time-dependent manner (Table 1). Aspirin, used as a reference drug, showed the highest latency (17.51 ± 0.50) at 1 h followed by reduction (16.82 ± 0.45) at 2 h. Among all the extracts of saffron tested, stigma ethanolic extract (SEE) showed the highest latency activity (7.80 ± 0.16, 11.30 ± 0.21, 12.80 ± 0.33, and 13.50 ± 0.28) at 0, 0.5, 1, and 2 h, respectively. On the other hand, leaf ethanolic extract (LEE) exhibited no significant difference at 0 h when compared with negative control (saline) but showed the least activity of 5.92 ± 0.14, 6.22 ± 0.15, and 6.65 ± 0.19 at 0.5, 1, and 2 h, respectively. Based on the percent inhibition (Figure 2), SEE was more prominent in reducing analgesia (44.91%, 63.89%, and 72.85%), followed by petal ethanolic extract (PEE), showing 38.84%, 53.07%, and 64.06% inhibition at 0.5, 1, and 2 h, respectively. Furthermore, corm ethanolic extract (CEE) and LEE responded poorly and showed weak analgesic activity of 22.40% and 29.07% at 2 h, respectively.

### 2.3. Carrageenan-induced Hind Paw Edema Test

**Figure 3:**
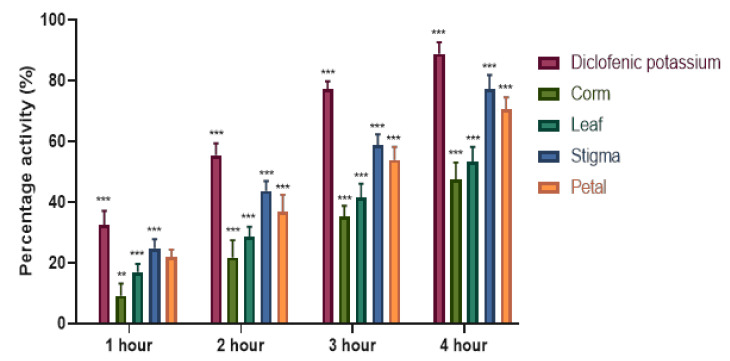
Percentage edema inhibition of C*. sativus* ethanolic extracts at selected time period in mice. Each value is represented as mean ± S.D. Where, * *p* < 0.05, ** *p* < 0.01, *** *p* < 0.001 statistically significant relative to control (saline).

The anti-inflammatory effects of saffron ethanolic extracts was investigated by the mouse paw-edema test, and the findings are presented in Table 2. Sub planter injection of carrageenan gradually increased edema paw volume of the saline-treated animals. However, as a positive control, diclofenac potassium attenuated paw edema volume by 88.87%, as depicted in Figure 3. Moreover, oral administration of saffron ethanolic extracts showed a significant (*p* < 0.05) decrease in edematous paw volume in a time-dependent manner. SEE (800 mg/kg) produced an anti-inflammatory activity 1 h after administration and continued until the end of the experimentation, with the most prominent inhibition of 77.33% followed by PEE (70.50%) at the fourth h of the study. LEE and CEE exhibited moderate but significant (*p* < 0.05) potential with the percentage edema inhibition of 53.29% and 47.47%, respectively.

### 2.4. Anticoagulant Assay

**Figure 4:**
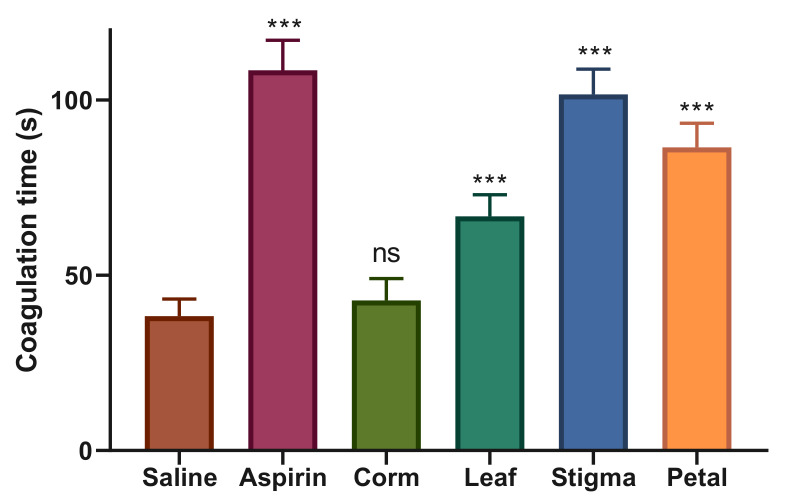
Anticoagulant effect of *C. sativus* ethanolic extracts. Each value is represented as mean ± S.D. Where, * *p* < 0.05, ** *p* < 0.01, *** *p* < 0.001 statistically significant relative to control (saline).

The blood clotting activity of saffron extracts was investigated using the capillary tube method. The results presented in Figure 4 show the effects of oral administration of saffron extracts and aspirin on coagulation time in mice. As a reference drug, the anticoagulant aspirin (10 mg/kg) significantly increased blood clotting time (108.5 ± 8.59 s) compared to the control group, saline (38.33 ± 4.92 s). SEE was the major identified anticoagulant extract showing prominent and significant anticoagulant effect with coagulation time of 101.66 ± 7.20 s followed by PCC (86.50 ± 6.89 s), respectively. LEE, however, had a moderate inhibitory effect on coagulation activity with a clotting time of 66.83 ± 6.17 s. CEE, on the other hand, was unable to prolong the blood clotting time (42.83 ± 6.27 s) and showed an almost equal response to saline, representing the neutral effect of the selected extract.

### 2.5. Antidepressant Activity

**Figure 5:**
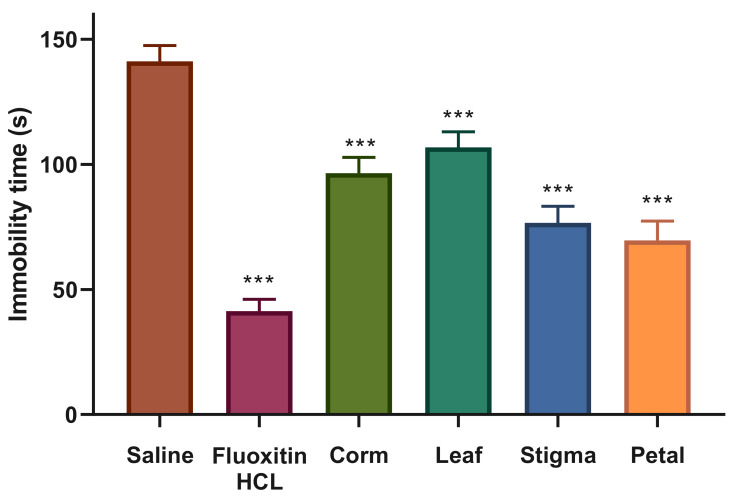
Antidepressant effect of *C. sativus* ethanolic extracts. Each value is represented as mean ± S.D. Where, * *p* < 0.05, ** *p* < 0.01, *** *p* < 0.001 statistically significant relative to control (saline).

The antidepressant activity of orally administered saffron ethanolic extracts was tested by forced swimming test, and findings in the form of immobility time are graphically depicted in Figure 5. Positive control group administered with drug, fluoxetine HCl produced a strong antidepressant effect (41.33 ± 4.71 s) at the concentration of 10 mg/kg against the negative control, saline (141.16 ± 6.40 s). Furthermore, saffron ethanolic extracts significantly attenuated immobility time in mice when compared with the saline-treated control group. At the dose of 800 mg/kg, PEE represented itself as a potential antidepressant candidate showing a significant reduction in immobility time (69.66 ± 7.63 s) with respect to control and equivalent to the standard drug followed by SEE (76.66 ± 6.56 s). CEE significantly declined the mean time spent by mice in the immobile state (96.50 ± 6.28 s), signifying moderate antidepressant effect, whereas LEE exhibited mild activity (106.83 ± 6.24 s) by significantly attenuating immobility time with respect to serine, but not equivalent to fluoxetine HCl.

## 3. Discussion

Saffron is an extraordinarily rich source of nutraceutical and pharmaceutical properties that exhibit numerous health benefits and pharmacological effects [42]. In the present study, we have evaluated the analgesic, anti-inflammatory, anticoagulant, and antidepressant activities of saffron ethanolic extracts in mice. The selection of 80% ethanol and/or ethanolic extracts was chosen for the study due to their efficiency against cancer [43,44], blood pressure [45,46], inflammation [47], and nociception [48,49]. None of the orally administered saffron extracts (800 mg/kg) caused any mortality or prominent behavioral abnormalities in mice during the 7-days acute toxicity study. In literature, toxicological reports regarding saffron safety are not uniform. Iranshahi et al. [50] assessed the toxicity level of 800 mg/kg SEE, PEE, stigma aqueous extract (SAE), and petal aqueous extract (PAE) and found no toxicological signs on mice. In another study, the maximum non-toxic dose of SEE and PEE was reported as 2 and 8 g/kg, respectively [48]. Furthermore, LD_50_ values of intraperitoneal administered petal and stigma extracts in mice were 6 and 1.6 g/kg, respectively [51]. Similarly, no mortality of mice was reported within 2 days of study with high oral and intraperitoneal doses (3 g/kg) of the active constituent of saffron, crocin, in mice [52].

The hot plate is one of the oldest and frequently used animal models to quantify “pain-like” behaviors in rodents [53]. Based on the species and strain of rodents used in clinical studies, nearly 12 different behaviors, including grooming, freezing, sniffing, licking, stamping of the legs, leaning, and jumping, have been measured in the hot plate assay [54]. In the present study, hot plate analgesic assay of saffron extracts demonstrated a time-dependent activity. Among all the saffron extracts, SEE exhibited the highest analgesic value in delaying the mean paw licking time (13.50 ± 0.28 s) by suppressing nociception in paws. PEE also delayed the onset time of licking response (11.13 ± 0.35 s). However, CEE and LEE exhibited weak analgesic activity of 22% and 29% at 2 h, respectively. Good analgesic drugs suppress the activity of nociceptors and exhibit the least number of lickings in animals. As per findings, SEE, and PEE thus were found as potent analgesic agents. The maximum analgesic activity of SEE might be due to the presence of carotenoids such as crocetin, crocin, picrocrocin, and safranal, as carotenoids have been reported to suppress the synthesis of prostaglandin synthetase [55]. This speculation is supported by another study where 0.1 and 0.2 g/kg crocin showed significant anti-edematogenic potential in histamine-induced paw edema in rats [56]. However, according to Hosseinzadeh and Younesi [48], aqueous and ethanolic stigma and petal extracts of saffron at any dose exerted no significant analgesic effect in mice. They suggested that the extracts might not act through central mechanisms, although drugs that alter the animal’s motor ability may enhance the licking duration on the hot plate method without affecting the central nervous system.

Inflammation serves as a body’s defensive biological response to damaged cells and injured tissue. The existence of edema is amongst the major signs of inflammation [57]. Carrageenan-induced paw edema is an established method to investigate the anti-edematous activity of natural products in rodents [58,59]. In the present study, the anti-inflammatory potential of saffron ethanolic extracts was studied after a sub-planter injection of 1% λ-carrageenan into mice. Test samples represented significant anti-edematous potential by regulating biphasic acute inflammatory response induced by carrageenan and showed the highest edema inhibition in mice after 4 h. Carrageenan induces the inflammatory process in two phases. The initial phase, which occurs during the first 2.5 h post-carrageenan injection, attributes to the release of mediators like serotonin, histamine, and bradykinin on vascular permeability. Serotonin and histamine are produced in the first 1.5 h, while bradykinin is produced within 2.5 h post-carrageenan injection [60]. The final phase occurs from 2.5 to 6 h after carrageenan injection, is associated with the overproduction of prostaglandins in tissues [58,61]. In addition to these mediators, Nitric Oxide (NO) is also reported to play a key role in carrageenan-induced acute inflammation [62,63]. Among all the extracts, SEE effectively inhibited the increase in paw volume of carrageenan-induced edema showing a maximum percentage of edema inhibition (77.33%) at the end of 4 h followed by PEE (70.50%) at 800 mg/kg dose. Diclofenac potassium used as a standard drug reduced paw edema volume by 88.87%. Therefore, it can be assumed that the active constituents of the extract might be responsible for the inactivation of inflammation process whose mechanism of action need to be studied. Phytochemicals screening of SAE, SEE, PAE, and PEE on acute inflammation by xylene-induced ear edema indicated that only SAE and SEE at higher doses possessed anti-inflammatory effects in mice. However, SAE, SEE, and PEE showed significant activity against chronic inflammation using formalin-induced edema in rat paw [48]. In another study, saffron aqueous extract suppressed formalin-induced paw edema in the chronic inflammation but failed to show activity against the acute phase of a formalin test [64]. Similarly, intraperitoneal injection of stigma constituent, crocin at concentrations of 0.1 and 0.2 g/kg significantly attenuated paw thickness and infiltration of neutrophils in paw tissues [56]. Kumar et al. [65] examined various petal extracts of *C. sativus* Cashmerianus to assess the anti-inflammatory effect by carrageenan-induced paw edema method. Among all the extracts, methanol extract (400 mg/mL) exhibited 63.16% inhibition of paw volume, followed by aqueous (57.89%) and chloroform (50%) extracts. The phytochemical profile of *C. sativus* petal suggests that the anti-inflammatory properties of PEE might be due to the presence of nutraceutical compounds, particularly flavonoids (kaempferol, 12.6% *w/w*) by modulating the gene and protein expression of inflammatory molecules [66].

The normal hemostatic process is meant to stop a cut or wound on blood vessels through platelet thrombus formation; subsequently, there is an eventual elimination of the plug when healing is complete. It is a delicate multiphase mechanism that requires the interaction of platelets and the coagulation factors with blood vessels. A defect in any of these phases can result in thrombosis or hemorrhage [67]. Extracts of various herbal plants have been used for their hemostatic role in wound healing, anti-infective, and antineoplastic properties [68]. Ankaferd Blood Stopper, an herbal extract from five plants (*Urtica dioica*, *Alpinia officinarum*, *Vitis vinifera*, *Thymus vulgaris*, and *Glycyrrhiza glabra*) is approved for the management of external hemorrhage and post-surgery dental bleedings in Turkey to attenuate blood clotting time effectively [69]. Additionally, crude extracts of *Fagonia cretica* (74.6%), *Hedera nepalensis* (73.8%)*,* and *Phytolacca latbenia* (67.3%) revealed promising anticoagulant effect by delaying blood clotting time to 86.9 s, 84.3 s, and 67.5 s, respectively [70]. In this study, saline and aspirin showed a clear line of difference between the coagulation and anticoagulation by depicting blood clotting time of 38.33 s and 108.5 s, respectively. The most significant anticoagulant effect was reported by SEE with a coagulation time of 101.66 s followed by PEE (86.5 s), respectively. LEE showed a blood clotting time of 66.83 s, representing a moderate inhibitory effect on coagulation activity. CEE was least effective in anticoagulation property and showed an almost equal response to saline, indicating a neutral effect of the selected extract, which can be used in the treatment of blood clotting disorders such as hemophilia. It is said that the corms of *C. sativus* Cartwrightianus have a protein factor involved in human platelet aggregation [71]. However, 5 years later, it was reported that it contains both activator/inducer of platelet aggregation [72]. Crocin delayed blood clotting time and mitigated respiratory distress as a result of pulmonary thrombosis in mice, inhibited thrombosis in rats, and suppressed platelet aggregation in rabbits [73]. Crocetin significantly reduced collagen- and ADP-induced platelet aggregation but failed to reduce arachidonic acid-induced platelet aggregation [74]. Besides that, crocetin significantly reduced dense granule secretion, while neither platelets adhesion to collagen nor cyclic AMP level was affected by crocetin [75]. Saffron stigma tablets (200 and 400 mg/day) assessed for short-term safety and tolerability in a limited number of volunteers showed that only 200 mg of saffron tablets reduced International Normalize Ratio, platelets, and coagulation time [76]. Later, in a double-blind, placebo-controlled clinical study with a large sample size, saffron tablets (200 and 400 mg/day) administration failed to show any major effect on coagulant and anticoagulant system after one month. The authors suggested that the case reports of hemorrhagic complications might be due to the high saffron dose, high period of consumption, or idiosyncrasy activities [77].

Depression is one of the most serious psychiatric disorders affecting approximately 4.7% of the global population and is ranked as the eleventh most frequent cause of disease burden worldwide [78]. Most of the patients have many concerns about commencing synthetic antidepressants in their recommended doses due to the anticipated adverse reactions such as libido, constipation, dry mouth, and dizziness [79]. Hence, extracts of medicinal plants provide the most effective sources of novel drugs showing promising results with minimum side effects in the routine treatment of depression [80,81]. The Forced Swimming Test (FST) or Porsolt swim test, is the most frequently used test to screen antidepressants among all rodents with more reliability, sensitivity, and specificity [82]. Immobility or the flouting response of rodents in FST is traditionally considered an indication of depression and anxiety [83]. During the FST, treatment of antidepressant drugs attenuate immobility, prolong its onset and delay active escape behaviors of the animal [84]. In the present study, a prominent difference in immobility time of saline (141.16 s) and fluoxetine HCl (41.33 s) used as a negative and positive control, respectively, was observed, representing the reliability of the test. Furthermore, the administration of saffron extracts showed a significant effect on reducing the immobility time compared to the saline-treated group. PEE represented itself as a potential antidepressant drug showing a notable diminution in the immobility period (69.66 s) followed by SEE (76.66 s). Moreover, CEE (96.50 s) indicated moderate antidepressant effect, whereas LEE (106.83 s) exhibited mild activity. The potent antidepressant effect exhibited by petal can be strongly linked to the presence of natural flavonoid, kaempferol as it significantly attenuated immobility behaviors in rats and mice and showed an almost similar response to fluoxetine [85]. Similarly, in an 8-week double-blind, randomized clinical study, dried saffron petal (15 mg bid) had similar antidepressant effects as fluoxetine (15 mg bid) in treating patients with mild-to-moderate depression and no significant differences in observed side effects [34]. In an investigation comparing the efficacy of saffron stigma and corms with fluoxetine against depression, petroleum ether, and dichloromethane fractions of saffron stigma and corms significantly reduced the immobility time in the tail suspension and forced swimming test at all doses (150, 300, and 600 mg/kg) without altering the locomotor behavior of mice during the open-field test. The authors highlighted that the antidepressant effect of stigma extracts could be due to crocin analogs, particularly crocin 1. However, HPLC analysis of corm extract revealed the absence of safranal, crocin, crocetin, or kaempferol compounds, assuming the presence of other bioactive compounds in saffron corms showing a potent antidepressant effect which need to be further explored [80]. The promising antidepressant activity of saffron secondary metabolites opens the doors for further studies to understand the mode of action in medicinal plants.

## 4. Materials and Methods

### 4.1. Plant Material

Saffron corms obtained from the safranor company, France, were planted in plastic trays and pots containing black soil in August. The soil texture of experimental black soil was a clay-loam structure containing 2.74% of total organic carbon and 0.04% of total nitrogen, in addition to 7 ppm of phosphorus, 331.5 ppm of potassium, 64 ppm of calcium, 75.6 ppm of magnesium, and 25.3 ppm of sodium. The corms were incubated in a temperature-controlled (18 ± 1 °C) room under 16 h photoperiod illuminated with white fluorescent tubes at an irradiance of nearly 40 µmol m^−2^ s^−1^. Different saffron parts were collected as per life span of the plant, i.e., petal and stigma were handpicked during October, leaves were collected in May, and smaller corms weighing less than 2 g were harvested in June. Individual parts were air-dried under shade for 7 weeks and ground to powder.

### 4.2. Chemicals

For animal experiments, all the chemicals, including aspirin, diclofenac potassium, fluoxetine HCl, ethanol, and carrageenan were purchased from Sigma. All chemicals used in the experiment were analytical grade.

### 4.3. Sample Extraction

Powder samples were macerated in ethanol/water (80%, *v/v*) for a week at room temperature. The mixture was subsequently filtered with Whatman filter paper 1 (Sigma-Aldrich, St. Louis, MO, USA) and concentrated by rotary evaporator under reduced pressure at 45 °C. Semi-liquid extracts were further allowed to dry in the fume hood. The crude extracts were freeze-dried and stored in sealed tubes at −20 °C for in vivo studies.

### 4.4. Standard and Test Drugs Preparation

Ethanolic extracts of saffron were prepared as 0.08 g/mL in 10% DMSO while standard drugs were dissolved in saline water (0.01 g/mL of 0.9% NaCl) and treated orally at 1 mL/100 g of mouse body weight. One optimal dose (800 mg/kg) showing maximum effect with no lethal consequences to mice was selected in this study, as reported previously [50].

### 4.5. Animals

Adult albino mice (Swiss strain) of either sex between 30–40 g were provided by Veterinary Research Institute Peshawar, Pakistan. All the mice were housed in aluminum cages (grade 304) under controlled natural 12/12 h light/dark cycle, temperature (21 ± 2 °C), and humidity (50–60%), and received tap water ad libitum and standard diet at the Animal House, Quaid-I-Azam University (QAU) Islamabad, Pakistan. Mice were acclimatized in this environment for seven days before the experiments. All the experiments were carried out between 10:00 and 17:00. The study protocol (Bch 0275) for laboratory animals was in accordance with the recommendations of the Institutional Animal Ethics Committee (QAU Islamabad), and all the precautionary measures were followed to reduce animal’s fear and suffering.

### 4.6. Study Design

Mice were weighed, marked with numbers, and split into 6 groups, each containing 6 mice. All the samples were administered by oral gavage. Group 1 received saline (0.9%) and was used as a negative control. Mice in group 2 were given a dose of 10 mg/kg standard drugs (aspirin for analgesic and anticoagulant, diclofenac potassium for anti-inflammatory, and fluoxetine HCl for antidepressant assay) used as a positive control. Group 3, 4, 5, and 6 were administered with a dose of 800 mg/kg CEE, LEE, SEE, and PEE, respectively.

#### 4.6.1. Acute Toxicity Study

The acute toxicity study of each tested sample was undertaken in accordance with the guidelines of the Organization for Economic Cooperation and Development (OECD). Albino mice (n=6) were administered orally with 800 mg/kg of each extract and observed for any changes in behavior and mortality at intervals of 0, 5, 10, 15, 20, and 24 h. Mice were examined for an additional 6 days for any signs of late morbidity and mortality.

#### 4.6.2. Hot Plate Analgesic Assay

The test first reported by Eddy and Leimbach [86], with some modifications, was performed to measure the analgesic activity of saffron extracts. The standard drug, aspirin (10 mg/kg), and saline (1 mL/kg) were served as a positive and negative control, respectively. The parameter recorded was based on the latency time for fore- and hind-paw licking and/or jumping responses by placing the mice on the surface of a hot-plate (IITC Life Science, USA) set at a temperature of 55 ± 1 °C and initial latency time (Ti) was calculated by taking the mean of two readings. Animals with baseline latencies of <5 s or >30 s were ignored from the study. The final latency time (Tf) was noted after administration of the drug for each group at the intervals of 0.5, 1, and 2 h, with a cut off time of 30 s. The tested extracts (0.8 g/kg) were administered orally, and standard (aspirin, 10 mg/kg) was administered subcutaneously. The percentage analgesia (PA) was measured using the equation:

(1) $$\mathrm{PA}\;=\;\left.\left(\;\frac{\mathrm{Tf}-\mathrm{Ti}}{\mathrm{Ti}}\right.\right)\;\times \;100$$

#### 4.6.3. Carrageenan-induced Paw Edema Test

*C. sativus* ethanolic extracts were examined for their anti-inflammatory activities using the carrageenan-induced paw edema test as described by Winter et al. [87]. The test is based on the principle to assess acute anti-inflammatory activity in the hind paws of mice by developing inflammatory models with carrageenan, a sulfated polysaccharide agent that triggers inflammation. In this test, diclofenac potassium (10 mg/kg) was used as a positive control, and saline (1 mL/kg) served as a negative control to compare the anti-inflammatory effects. Before experimentation, mice were fasted for 24 h but had access to water. For edema induction, 1% λ-carrageenan (0.1 mL), prepared in 1% saline was injected into the sub planter tissue of mice left hind paw 1 h after administration of the drug. The basal volume of the paw was recorded just before and after injection of λ-carrageenan at 0, 1, 2, 3, and 4 h by digital plethysmometer (UGO Basile, 7140). For every interval, all the data were recorded in triplicate. The degree of swelling was calculated by the delta volume (A−B), where “A” shows the mean volume of the left hind paw after and “B” before the treatment of *λ*-carrageenan. The percentage edema inhibition (PEI) was measured using the equation:

(2) $$\mathrm{PEI}\;=\;\left.\left(\;\frac{A-B}{A}\right.\right)\;\times \;100$$

#### 4.6.4. Anticoagulant

Blood clotting activity of saffron extracts was evaluated by the capillary tube method reported by Ismail and Mirza [84]. One hour after oral administration of the dose, the mouse tail was disinfected with spirit and prickled using a lancet. The tail was pressed firmly to get a bigger drop of blood and collected in capillary tubes. The time of appearance of the blood drop on the cut tail was recorded. The tubes were wrapped and maintained at 37 ± 1 °C in a water bath. Small portions of each tube were broken at regular intervals of 10 s, until fibrin thread appeared. The blood coagulation time was measured by considering the appearance of a blood drop as a starting point and thread formation as an endpoint.

#### 4.6.5. Forced Swimming Test

The forced swim test (FST) described by Porsolt et al. [88] was used to screen the extracts of saffron for their antidepressant activity. On day first, mice were forced to swim individually in a glass tank of 40 cm height and 18 cm diameter containing water up to 30 cm under natural light. The water temperature was adjusted to 25 ± 1 °C. The water level was adjusted in such a way that mice could only touch the bottom of the tank with the tip of the tail. After 15 min exposure in the tank, mice were evacuated, dried off with a paper towel, and shifted to their home cage. The next day, saline (1 mL/kg) and fluoxetine HCl (10 mg/kg) served as a negative and positive control, respectively, along with saffron extracts (800 mg/kg) were administered 30 min before the experiment. Mice were allowed to swim freely for 6 min in the tank, and swimming behaviors were recorded with a video camera. Before each test, freshwater was introduced. Animals were placed one by one, and after every 2 min, immobility time was calculated in the last 4 min of swimming practice using a stopwatch. Immobility time is the time when the mouse stopped all additional movements other than those required for survival or balancing of their body.

### 4.7. Statistical Analysis

Data were expressed as mean along with standard deviation. Significant difference between groups was analyzed with One-way ANOVA in anticoagulant and antidepressant assays and two-way ANOVA in analgesic and anti-inflammatory assays followed by the Post Hoc Dunnett test. Graphs were made in GraphPad Prism 5.0 (La Jolla, CA, USA). The results were considered to be significant at *p* < 0.05.

## 5. Conclusions

To the best of our knowledge this is the first report of CEE, LEE, SEE, and PEE from in vivo grown *Crocus sativus* L. to determine anti-inflammatory, analgesic, anticoagulant, and antidepressant properties in mice. SEE and PEE were evinced as a safe natural remedy to treat pain, inflammation, depression, and the coagulation system. The above results clearly indicate that saffron is an excellent source of bioactive compounds having great potential as nutraceuticals and health benefits. These properties can be linked to intrinsic active compounds such as carotenoids and flavonoids found in the highest amounts in stigma and petals, respectively. However, further epidemiological investigations, laboratory research, and clinical trials are required to isolate the pharmacologically active molecules that contribute to the observed effects and to explicate the possible mechanism of action and effect of the plant on various critical illnesses and medicinal formulations.

## Figures and Tables

**Table 1:** Latency time of *C. sativus* ethanolic extracts in hot plate assay.

Group	Dose (mg/kg)	Latency Time (s)			
		0 h	0.5 h	1 h	2 h
Saline	1 mL/kg	4.70 ± 0.06	5.00 ± 0.02	5.50 ± 0.01	5.40 ± 0.04
Aspirin	10	9.45 ± 0.28^***^	16.38 ± 0.42^***^	17.51 ± 0.50^***^	16.82 ± 0.45^***^
CEE	800	6.20 ± 0.17^***^	6.93 ± 0.16^***^	7.15 ± 0.13^***^	7.58 ± 0.14^***^
LEE	800	5.15 ± 0.15	5.92 ± 0.14^**^	6.22 ± 0.15^*^	6.65 ± 0.19^**^
SEE	800	7.80 ± 0.16^***^	11.30 ± 0.21^***^	12.80 ± 0.33^***^	13.50 ± 0.28^***^
PEE	800	6.78 ± 0.17^***^	9.42 ± 0.24^***^	10.38 ± 0.29^***^	11.13 ± 0.35^***^

Each value is represented as mean ± S.D. Where, *p < 0.05, ** *p* < 0.01, *** *p* < 0.001 statistically significant relative to control (saline).

**Table 2:** Anti-inflammatory effect of *C. sativus* ethanolic extracts.

Group	Dose (mg/kg)	Mean Edema Volume (mL)			
		1 h	2 h	3 h	4 h
Saline	1 mL/kg	0.47 ± 0.03	0.56 ± 0.03	0.69 ± 0.04	0.76 ± 0.03
Diclofenac potassium	10	0.25 ± 0.02^***^	0.17 ± 0.01^***^	0.08 ± 0.01^***^	0.04 ± 0.01^***^
CEE	800	0.43 ± 0.03	0.37 ± 0.03^***^	0.30 ± 0.01^***^	0.25 ± 0.03^***^
LEE	800	0.37 ± 0.03^**^	0.31 ± 0.02^***^	0.26 ± 0.01^***^	0.20 ± 0.03^***^
SEE	800	0.26 ± 0.01^***^	0.19 ± 0.02^***^	0.14 ± 0.01^***^	0.07 ± 0.01^***^
PEE	800	0.29 ± 0.02^***^	0.23 ± 0.02^***^	0.17 ± 0.01^***^	0.11 ± 0.01^***^

Each value is represented as mean ± S.D. Where, *p < 0.05, ** *p* < 0.01, *** *p* < 0.001 statistically significant relative to control (saline).
